# Supplementary material for: Translational repression by 4E-T is crucial to maintain the prophase-I arrest in vertebrate oocytes
Source: Nat Commun. 2025 Aug 28;16:8051. doi: 10.1038/s41467-025-62971-9 (PMC12394692; doi:10.1038/s41467-025-62971-9)
Supplement: Supplementary file 1 — Supplementary Information [file 41467_2025_62971_MOESM1_ESM.pdf]

Supplementary Figure 1

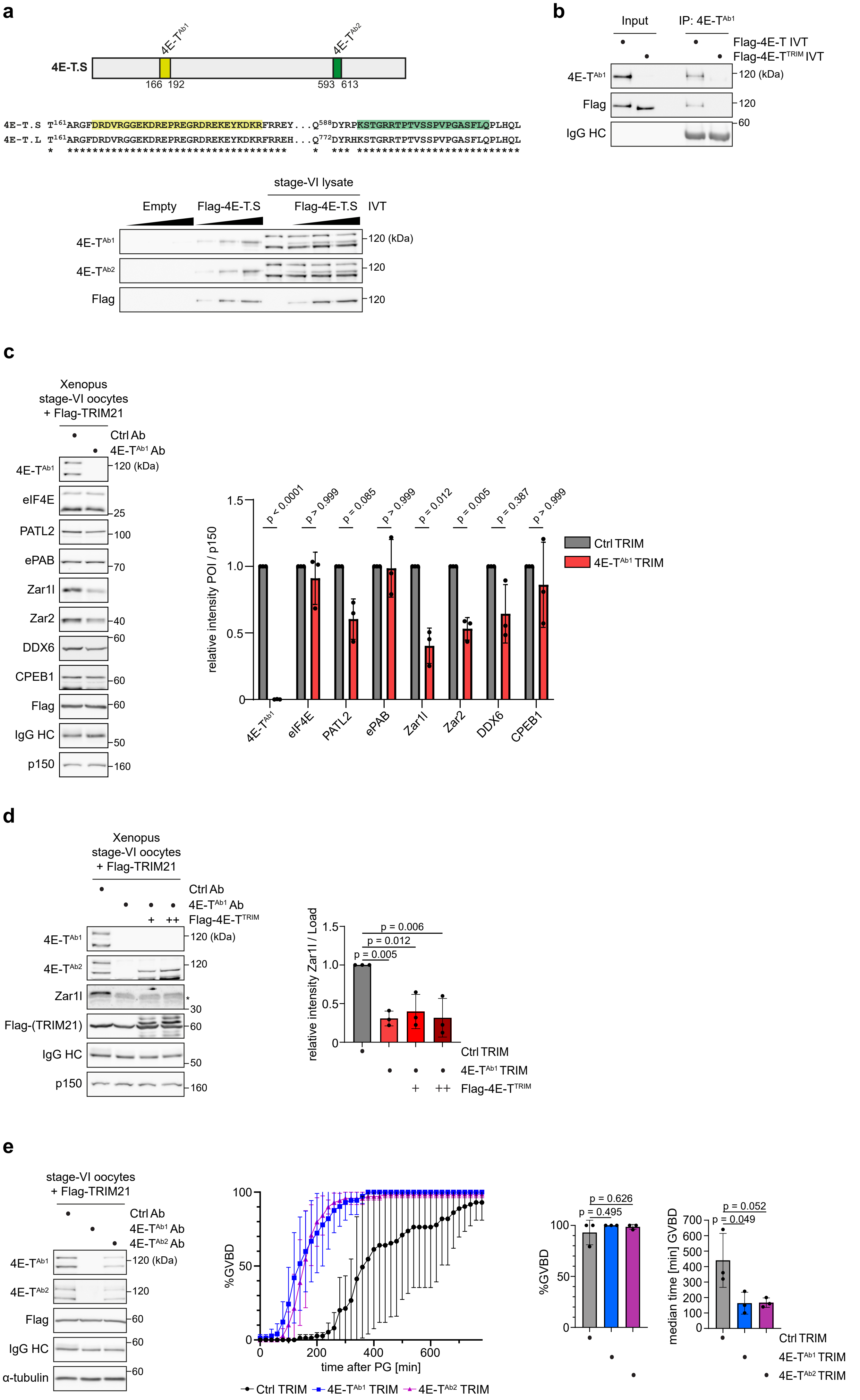

Supplementary Fig. 1

a) Schematic representation of 4E-T.S from *Xenopus laevis*. The antigens used for the generation of 4E-T^{Ab1} and 4E-T^{Ab2} are highlighted in yellow and green, respectively. *In vitro* translation reactions (IVT) of Flag-4E-T.S were immunoblotted as indicated. An IVT reaction not expressing a specific protein (Empty IVT) was loaded as a control. Where indicated Flag-4E-T.S IVT was mixed with lysate of Xenopus stage-VI oocytes.

b) IVT reactions expressing Flag-4E-T or Flag-4E-T^{TRIM} were subjected to immunoprecipitation with 4E-T^{Ab1}. Input and IP samples were immunoblotted as indicated.

c) Xenopus stage-VI oocytes were injected with 4E-T^{Ab1} or unspecific control (Ctrl) antibodies and mRNA encoding Flag-TRIM21. 22h after injection, oocytes were lysed and analyzed by immunoblotting. Protein signals were quantified and normalized to p150. Values were normalized to the Ctrl TRIM condition and are given as mean±s.d. from three independent biological replicates. p-values were calculated using unpaired two-sided t-test with Bonferroni-Dunn correction for multiple comparisons.

d) Xenopus stage-VI oocytes were injected with water or mRNA encoding Flag-4E-T^{TRIM}. 18h after injection, oocytes were co-injected with mRNA encoding Flag-TRIM21 and either 4E-T^{Ab1} or unspecific control (Ctrl) antibodies. 22h after the second injection, oocytes were lysed for immunoblotting as indicated. Zar1l signals were quantified and normalized to load. Values were normalized to the Ctrl TRIM condition and are given as mean±s.d. from three independent biological replicates. p-values were calculated using one-way ANOVA with Tukey's multiple comparisons test.

e) Xenopus stage-VI oocytes were injected with the indicated 4E-T or unspecific control (Ctrl) antibodies and mRNA encoding Flag-TRIM21. 22h after injection, some oocytes were lysed for immunoblotting. The residual oocytes (Ctrl TRIM, n=74 oocytes; 4E-T^{Ab1} TRIM, n=73 oocytes; 4E-T^{Ab2} TRIM, n=69 oocytes) were treated with PG and time until GVBD was determined. In addition, percentage of oocytes undergoing GVBD in 780min after PG addition and median time to GVBD were quantified. All values are given as mean±s.d. from three independent biological replicates. p-values were calculated using one-way ANOVA with Tukey's multiple comparisons test.

Source data including additional loading controls are provided as a Source Data file.

Supplementary Figure 2

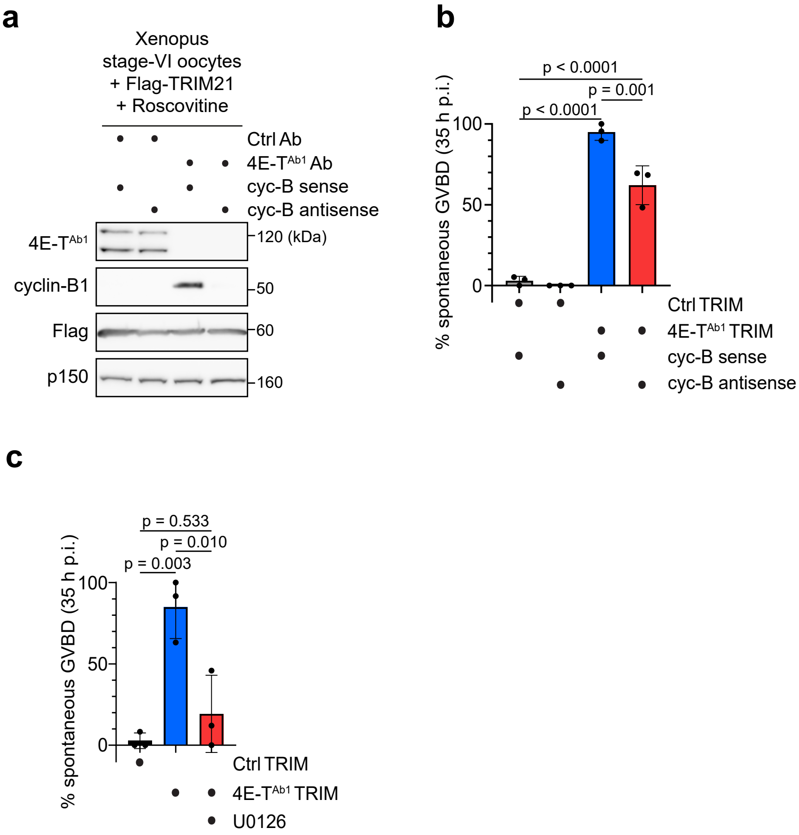

Supplementary Fig. 2

a) Xenopus stage-VI oocytes were co-injected with mRNA encoding Flag-TRIM21, 4E-T^{Ab1} or unspecific control (Ctrl) antibodies and cyc-B sense or antisense oligonucleotide mix. Oocytes were incubated in medium containing the Cdk inhibitor Roscovitine. 48h after injection, oocytes were lysed and immunoblotted as indicated. One representative experiment of three independent biological replicates is shown.

b) Xenopus stage-VI oocytes were co-injected with mRNA encoding Flag-TRIM21, 4E-T^{Ab1} or unspecific control (Ctrl) antibodies and cyc-B sense or antisense oligonucleotide mix. 35h after injection, the occurrence of GVBD was determined by the appearance of a white spot in the animal hemisphere of the oocytes (Ctrl TRIM + cyc-B sense, n=69 oocytes; Ctrl TRIM + cyc-B antisense, n=68 oocytes; 4E-T^{Ab1} TRIM + cyc-B sense, n=69 oocytes; 4E-T^{Ab1} TRIM + cyc-B antisense, n=71 oocytes). Percentage of oocytes with GVBD spots is given as mean±s.d. from three independent biological replicates. p-values were calculated using one-way ANOVA with Tukey's multiple comparisons test.

c) Xenopus stage-VI oocytes were co-injected with mRNA encoding Flag-TRIM21 and 4E-T^{Ab1} or unspecific control (Ctrl) antibodies. As indicated, oocytes were treated with the MEK inhibitor U0126. 35h after injection, the occurrence of GVBD was determined by the appearance of a white spot in the animal hemisphere of the oocytes (Ctrl TRIM + DMSO, n=69 oocytes; 4E-T^{Ab1} TRIM + DMSO, n=69 oocytes; 4E-T^{Ab1} TRIM + U0126, n=69 oocytes). Percentage of oocytes with GVBD spots is given as mean±s.d. from three independent biological replicates. p-values were calculated using one-way ANOVA with Tukey's multiple comparisons test.

Source data including additional loading controls are provided as a Source Data file.

Supplementary Figure 3

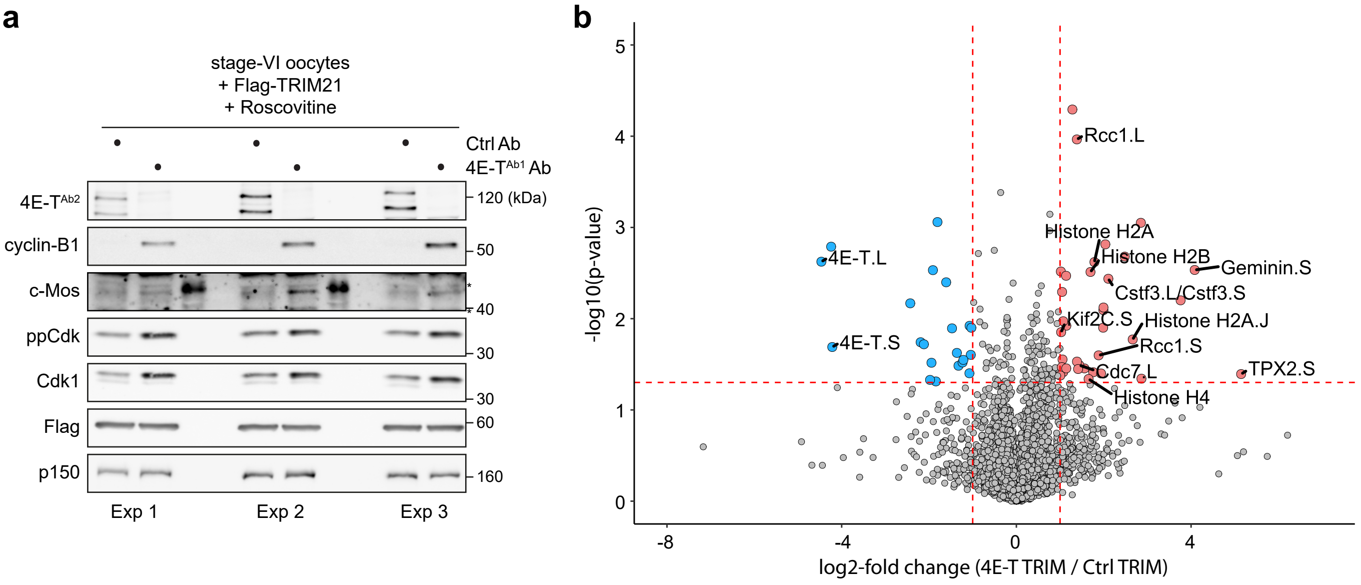

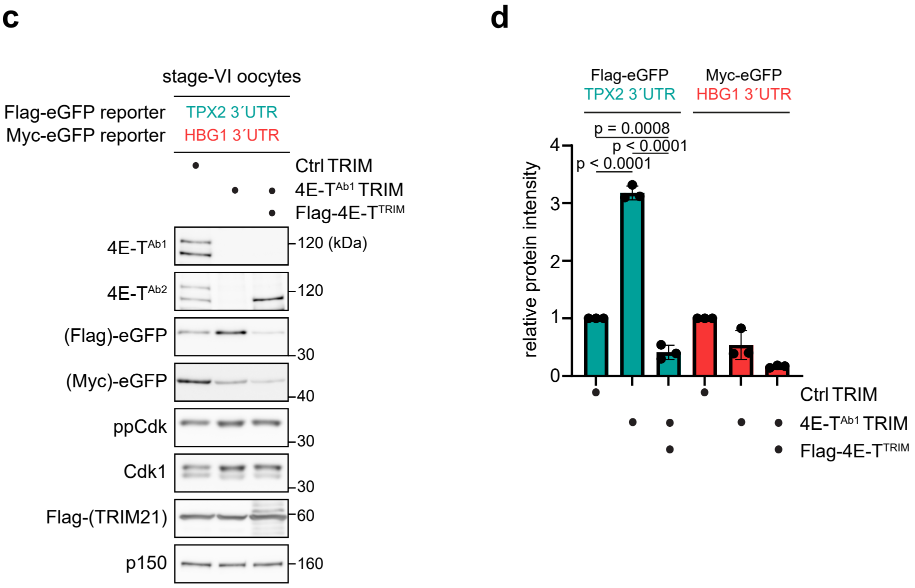

Supplementary Fig. 3

a) Xenopus stage-VI oocytes were co-injected with mRNA encoding Flag-TRIM21 and 4E-T^{Ab1} or unspecific control (Ctrl) antibodies. Oocytes were incubated in medium containing the Cdk inhibitor Roscovitine. 42h after injection, oocytes were lysed and immunoblotted as indicated. Three independent biological replicates are shown. Asterisks indicate unspecific bands.

b) Proteins in oocyte lysates of all three biological replicates from a) were identified using LC-MS/MS analysis. Volcano plot shows difference in protein expression between 4E-T TRIM and Ctrl TRIM conditions. Selected significantly enriched proteins (log2-fold change >1 and p <0,05 as determined by an unpaired Student's t-test (n=3)) are highlighted in red (enriched in 4E-T TRIM) and blue (depleted in 4E-T TRIM), respectively.

c) Xenopus stage-VI oocytes were injected with water or mRNA encoding Flag-4E-T^{TRIM}. 18h after injection, oocytes were co-injected with mRNA encoding Flag-TRIM21, with 4E-T^{Ab1} or unspecific control (Ctrl) antibodies, with mRNA encoding Myc-eGFP_HBG1 3´UTR and with mRNA encoding Flag-eGFP_TPX2 3´UTR. Oocytes were incubated in medium containing the Cdk inhibitor Roscovitine, lysed after 22h and immunoblotted as indicated. Asterisks indicate unspecific bands. One representative experiment of three independent biological replicates is shown.

d) eGFP signals in c) were quantified and normalized to p150. Values were normalized to the Ctrl TRIM condition and are given as mean±s.d. from three independent biological replicates. p-values were calculated using one-way ANOVA with Tukey's multiple comparisons test.

Source data including additional loading controls are provided as a Source Data file.

Supplementary Figure 4

a

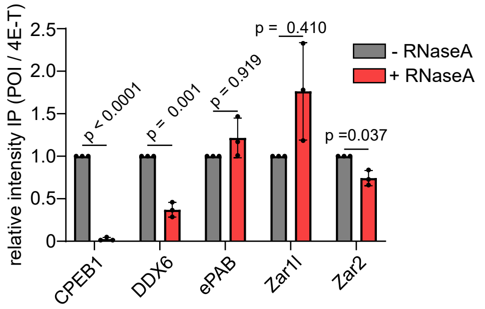

b

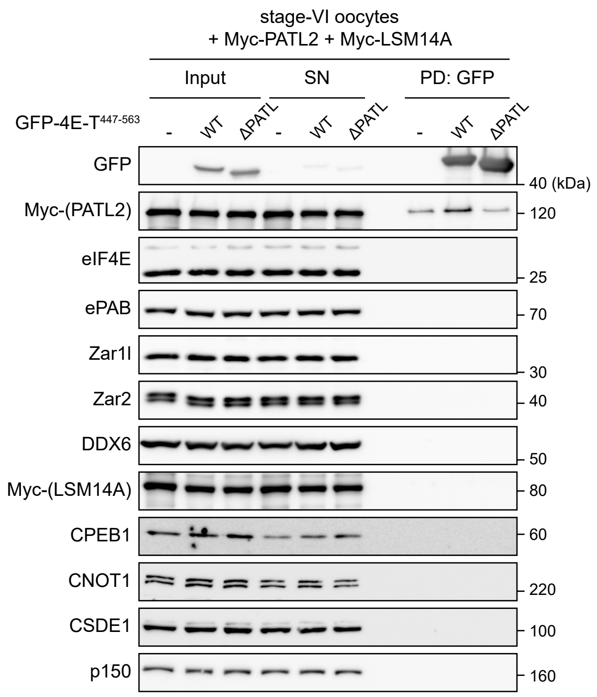

Supplementary Fig. 4

a) Quantification of Fig. 3a. Protein signals in IP samples were quantified. Signals in Ctrl IP samples were subtracted from signals in 4E-T^{Ab1} IP samples and values were normalized to 4E-T. Values were normalized to IP without RNaseA and are given as mean±s.d. from three independent biological replicates. p-values were calculated using unpaired two-sided t-test with Bonferroni-Dunn correction for multiple comparisons.

b) Xenopus stage-VI oocytes were injected with water or mRNA encoding the indicated GFP-4E-$T^{447\text{-}563}$ variant. 18h after injection, oocytes were lysed and subjected to pull-down against the GFP-tag. Samples were immunoblotted as indicated. One representative experiment of three independent biological replicates is shown.

Source data including additional loading controls are provided as a Source Data file.

Supplementary Figure 5

a

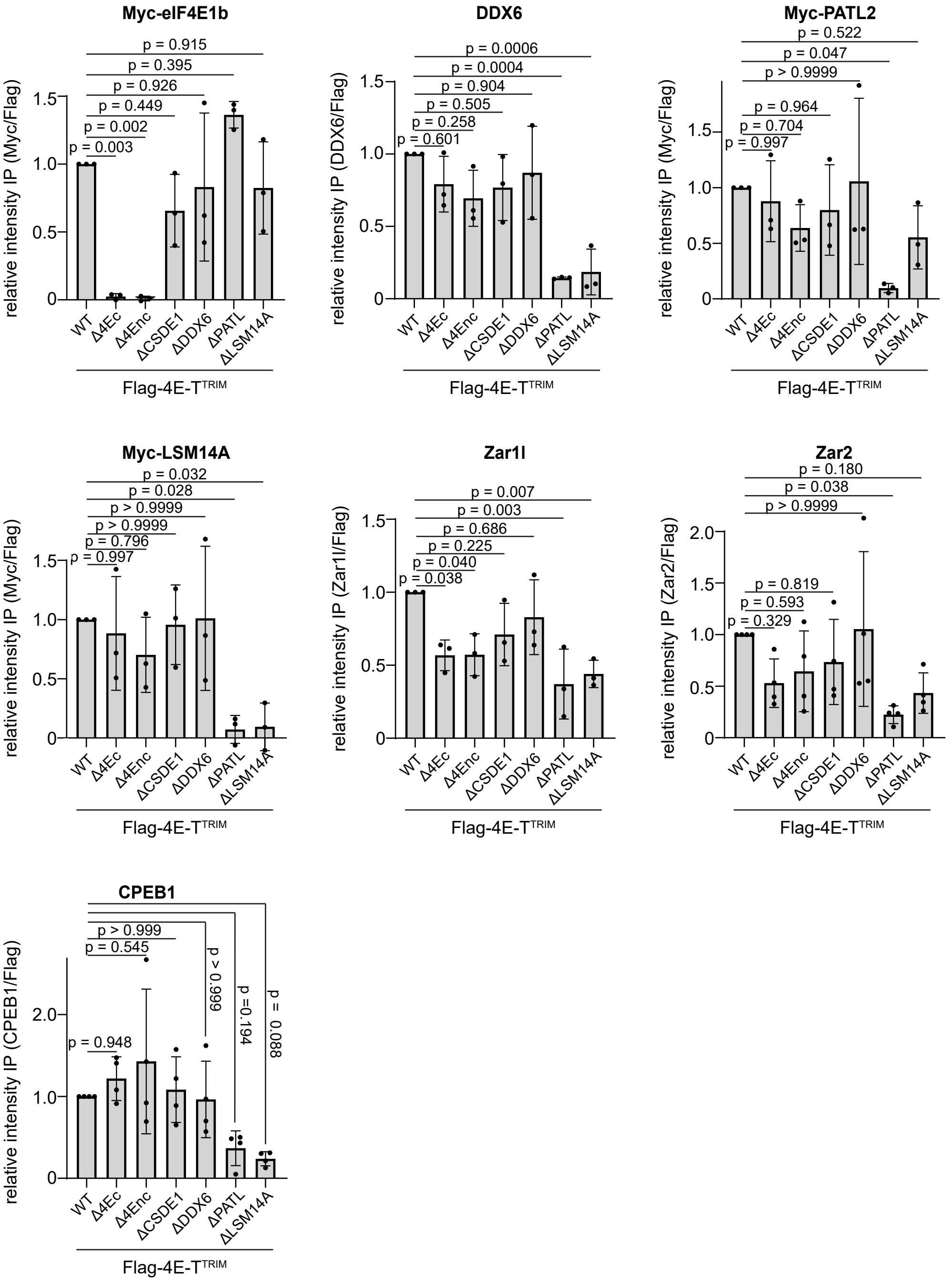

Supplementary Fig. 5

a) Quantification of Figs. 4b and 4c. Protein signals in IP samples were quantified. Signals in IP samples of water-injected oocytes were subtracted and values were normalized to Flag. All conditions were normalized to WT and values are given as mean±s.d. from three (for Myc-eIF4E1b, DDX6, Myc-PATL2, Myc-LSM14A and Zar1l) or four (for Zar2 and CPEB1) independent biological replicates. p-values were calculated using one-way ANOVA with Dunnett's multiple comparisons test.

Source data are provided as a Source Data file.

Supplementary Figure 6

a

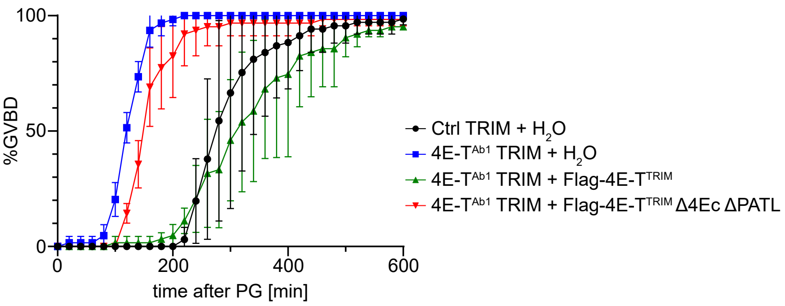

b

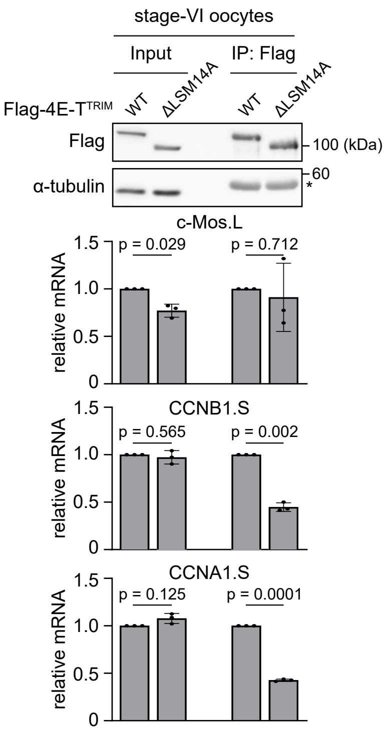

Supplementary Fig. 6

a) Additional quantification of GVBD timing from experiments in Fig. 5d.

b) Xenopus stage-VI oocytes were injected with mRNA encoding Flag-4E-T^{TRIM} WT or ΔLSM14A. 18h after injection, oocytes were lysed and subjected to immunoprecipitation with Flag antibodies. Samples were immunoblotted as indicated. Asterisk indicates IgG HC. In parallel, RNA was isolated from the same samples and analyzed by qRT-PCR for the indicated mRNAs. Values in input and IP samples were normalized to the Flag-4E-T^{TRIM} WT conditions and are given as mean±s.d. from three independent biological replicates. p-values were calculated using unpaired two-sided t-test with Welch´s correction.

Source data are provided as a Source Data file.

Supplementary Figure 7

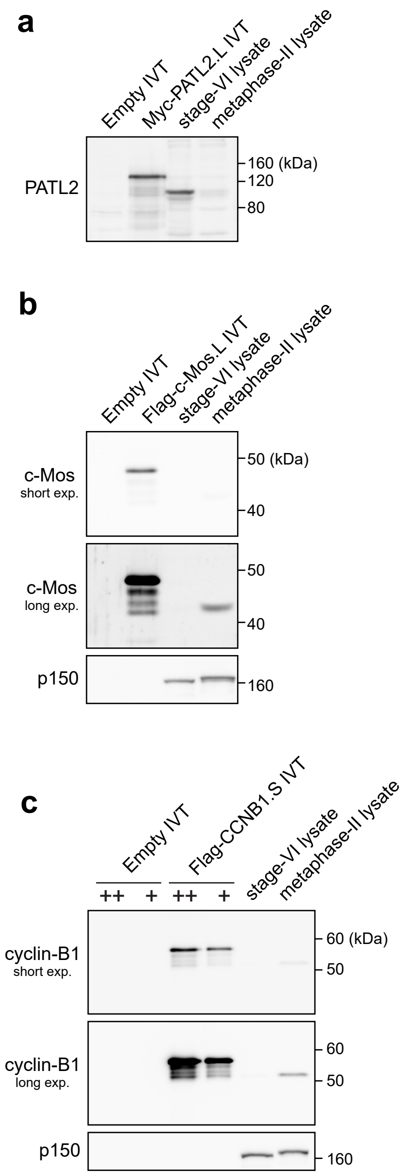

Supplementary Fig. 7

a) *In vitro* translation reactions (IVT) expressing Myc-PATL2.L or no specific protein (Empty) and lysates of Xenopus stage-VI or metaphase-II-arrested oocytes were immunoblotted as indicated.

b) *In vitro* translation reactions (IVT) expressing Flag-c-Mos.L or no specific protein (Empty) and lysates of Xenopus stage-VI or metaphase-II-arrested oocytes were immunoblotted as indicated.

c) *In vitro* translation reactions (IVT) expressing Flag-CCNB1.S or no specific protein (Empty) and lysates of Xenopus stage-VI or metaphase-II-arrested oocytes were immunoblotted as indicated.

Source data are provided as a Source Data file.

Supplementary Table 1. Oligonucleotide Primer Sequences

4E-T.S Fse1 fwd	ATTAGGCCGGCCCATGGATTATAGAGAAGAGACA GATC
4E-T.S Asc1 rev	TAATGGCGCGCCTCACTGTCTATACTCCAGTTCAT C
4E-T TRIM-res (Δ169-189) fwd	AGAGGATTTGACAGAGATGACAAACGCTTCAGGC GAGAATAT
4E-T TRIM-res (Δ169-189) rev	ATATTCTCGCCTGAAGCGTTTGTCATCTCTGTCAA ATCCTCT
4E-T Gln319 Asc1 rev	TAATGGCGCGCCTCATTGATTTCTTGAAGCCAGGC CTG
4E-T Δ4Ec (Δ28-34) fwd	GGCAGATCTCATCACAGTGACATCAAAGAACTTCC ACACTCC
4E-T Δ4Ec (Δ28-34) rev	GGAGTGTGGAAGTTCTTTGATGTCACTGTGATGAG ATCTGCC
4E-T Δ4Enc (Δ51-65) fwd	AGGCCCTCTTGTTTGCTTTCTTCCCTCTACCCAAA CTCAGGG
4E-T Δ4Enc (Δ51-65) rev	CCCTGAGTTTGGGTAGAGGGAAGAAAGCAAACAA GAGGGCCT
4E-T ΔCSDE1 (Δ130-160) fwd	CACGTTACAGCTGCTGCCACTGCAAGAGGATTTGA CAGAGAT
4E-T ΔCSDE1 (Δ130-160) rev	ATCTCTGTCAAATCCTCTTGCAGTGGCAGCAGCTG TAACGTG
4E-T ΔDDX6 (Δ218-239) fwd	TCTTGTACAGAGGAGGAAAAAATTCTGGAAGAGGA TCAGAAG
4E-T ΔDDX6 (Δ218-239) rev	CTTCTGATCCTCTTCCAGAATTTTTTCCTCCTCTGT ACAAGA
4E-T ΔPATL (Δ504-522) fwd	ACAAGCATGCTATCTCCAGATGAACCTACAGAAAA GCAAAAT
4E-T ΔPATL (Δ504-522) rev	ATTTTGCTTTTCTGTAGGTTCATCTGGAGATAGCAT GCTTGT
4E-T Met725 Asc1 rev	TAATGGCGCGCCTCACATGTTGGGGCGGTTTGTC
4E-T.S Gln447 Fse1 fwd	ATTAGGCCGGCCCCAAGCTGCCAACTACTATCAC
4E-T.S Gly563 Asc1 rev	TAATGGCGCGCCTCATCCCATCACCAGGGATGTG TC
PATL2.L Fse1 fwd	ATTAGGCCGGCCCATGAATCTCGGCTCCGAAC
PATL2.L Asc1 rev	TAATGGCGCGCCTCATGAAGGTACAGCTGTGTAT G
LSM14A.L Fse1 fwd	ATTAGGCCGGCCAATGAGCGGGGGTACTCC
LSM14A.L Asc1 rev	TAATGGCGCGCCCTAGGCTGCCACTTTGTTGTC
eIF4E1b.S Fse1 fwd	ATTAGGCCGGCCAATGGCAGCAGCTGAAGCATTA AG
eIF4E1b.S Asc1 rev	TAATGGCGCGCCTCAGACCACAAACTTGTTCTTGG A
c-Mos.L 3´UTR Xba1 fwd	ATTATCTAGACGTCCAGAACAGGGAGC
c-Mos.L 3´UTR+5A Xba1 rev	TAATTCTAGATTTTTAGACAAATCAATTTCTTTATTA TAAAACTATATATTCACATATG
CCNB1.S 3´UTR+5A Xba1 Oligo 1	ATTATCTAGAGACACTTGTTATATTGTAGAACATTT TTAACCAATGCTCTTACTGTGTATTTTATTATTTTAA TAAAGATTATTTTGAAAAATCTAGAATTA
CCNB1.S 3´UTR+5A Xba1 Oligo 2	TAATTCTAGATTTTTCAAAATAATCTTTATTAAAATA ATAAAATACACAGTAAGAGCATTGGTTAAAAATGTT CTACAATATAACAAGTGTCTCTAGATAAT
CCNA1.S 3´UTR Xba1 fwd	ATTATCTAGAAGCCTTCCAGAGTGGACG
CCNA1.S 3´UTR+5A Xba1 rev	TAATTCTAGATTTTTACCGTTTGAGTAAAGTCAGTT TATTAAAAAC
TPX2.S 3´UTR Xba1 fwd	ATTATCTAGATGTGCTCCCTGTACTAAGCAAATC

TPX2.S 3´UTR+5A Xba1 rev	TAATTCTAGATTTTTCAACTTTACATTTCCACAGTTT ATTACAG
Hbg1.L 3´UTR Xba1 fwd	ATTATCTAGAACCAGCCTCAAGAACACCC
Hbg1.L 3´UTR+5A Xba1 rev	TAATTCTAGATTTTTGTGAAGAAACTTTCTTTTTATT AGGAGCAG
Flag-eGFP_c-Mos 3´UTR PCR for mRNA template fwd	GCCATTCTGCCTGGGG
Flag-eGFP_c-Mos 3´UTR+30A PCR for mRNA template rev	TTTTTTTTTTTTTTTTTTTTTTTTTTTTTTAGACAAAT CAATTTCTTTATTATAAAACTATATATTCACATATG
Flag-eGFP_CCNB1 3´UTR PCR for mRNA template fwd	GCCATTCTGCCTGGGG
Flag-eGFP_CCNB1 3´UTR+30A PCR for mRNA template rev	TTTTTTTTTTTTTTTTTTTTTTTTTTTTTTCAAAATAAT CTTTATTAAAATAATAAAATACACAGTAAGAGC
Flag-eGFP_CCNA1 3´UTR PCR for mRNA template fwd	GCCATTCTGCCTGGGG
Flag-eGFP_CCNA1 3´UTR+30A PCR for mRNA template rev	TTTTTTTTTTTTTTTTTTTTTTTTTTTTTTACCGTTTG AGTAAAGTCAGTTTATTAAAAAC
Flag-eGFP_TPX2 3´UTR+30A PCR for mRNA template rev	TTTTTTTTTTTTTTTTTTTTTTTTTTTTTTCAACTTTAC ATTTCCACAGTTTATTACAG
Myc-eGFP_HBG1 3´UTR PCR for mRNA template fwd	GCCATTCTGCCTGGGG
Myc-eGFP_HBG1 3´UTR+30A PCR for mRNA template rev	TTTTTTTTTTTTTTTTTTTTTTTTTTTTTTGTGAAGAA ACTTTCTTTTTATTAGGAGCAG
Flag-eGFP reporter mRNA RT-PCR fwd	TGTTCTTTTTGCAGGATCCAC
Myc-eGFP reporter mRNA RT-PCR fwd	TCTTTTTGCAGGATCCCATC
Flag/Myc-eGFP reporter mRNA RT-PCR rev	GAACTTCAGGGTCAGCTTGC
λN-tag BamH1 fwd	ATTAGGATCCACCATGGACGCACAAACACGACG
λN-tag-Linker BamH1 rev	TAATGGATCCACCGGACCCACTTGTGCTACCCGAT CCCTTTGAGTTTGCAGCTTTCCATTGAGCTTGT
5xboxB 3´UTR Asc1 fwd	ATTAGGCGCGCCTAAGTCCAACTACTAAACTGGG GAT
5xboxB 3´UTR Xba1 rev	TAATTCTAGACATAATATCCTCGAGATAATATCCTC GATA
c-Mos.L qRT-PCR fwd	GAACCTACACTCACCGAGCC
c-Mos.L qRT-PCR rev	AGGCCACTACCGCATAGAGA
CCNB1.S qRT-PCR fwd	AGCTCTTCGGAAACCCACTG
CCNB1.S qRT-PCR rev	AGCTGGTTCTGGTTGCATCT
CCNA1.S qRT-PCR fwd	ATGAAGACACTGCAGGCCAA
CCNA1.S qRT-PCR rev	CCAGTCAGCAACTAGTGTCCA
TPX2.S qRT-PCR fwd	GTGCCTCAGTCTCCTGCTTT
TPX2.S qRT-PCR rev	AAGGGACAAGCCTCCACTTG
BTG4.L qRT-PCR fwd	GCCTTCAGACAGTCTTCTGCC
BTG4.L qRT-PCR rev	GCCATTGGCTAGTTTGGAGC
Wee2.L qRT-PCR fwd	AGGGAGTTGAAAGCCGCTAA
Wee2.L qRT-PCR rev	GCCCAGCGAGGAAAAGAAC
XErp1.L qRT-PCR fwd	GGGTTTATGGGTGGGGCTTT
XErp1.L qRT-PCR rev	TGGCATTAAGTGCTATGGTTGC
ACTA2.S qRT-PCR fwd	ACCAGAATACGACGAAGCCG
ACTA2.S qRT-PCR rev	TTTTGGAATGAAACGGTGGCG
Hbg1.L qRT-PCR fwd	GACAAGAGGCCCTTGGACG
Hbg1.L qRT-PCR rev	ATGTGCTTGATGGCCTCTCC
Hbg2.S qRT-PCR fwd	GGCTGCTCATGGTGAAAAGG
Hbg2.S qRT-PCR rev	AGTGGTGAGCCAGGGTAATG
cyc8 antisense	G*T*A*CATCTCTTCAT*A*T*T (* denotes phosphothioate)
cycB5-2 antisense	T*C*C*ATCTGTCCT*G*T*A (* denotes phosphothioate)

cyc8 sense	A*A*T*ATGAAGAGATG*T*A*C (* denotes phosphothioate)
cycB5-2 sense	T*A*C*AGGACAGAT*G*G*A (* denotes phosphothioate)
