# Supplementary material for: Translational repression by 4E-T is crucial to maintain the prophase-I arrest in vertebrate oocytes
Source: Nat Commun. 2025 Aug 28;16:8051. doi: 10.1038/s41467-025-62971-9 (PMC12394692; doi:10.1038/s41467-025-62971-9)
Supplement: Supplementary file 2 — Reporting Summary [file 41467_2025_62971_MOESM2_ESM.pdf]

nature portfolio

Corresponding author(s): Andreas Heim and Thomas U. Mayer

Last updated by author(s): Jul 23, 2025

Reporting Summary

Nature Portfolio wishes to improve the reproducibility of the work that we publish. This form provides structure for consistency and transparency in reporting. For further information on Nature Portfolio policies, see our Editorial Policies and the Editorial Policy Checklist.

Statistics

For all statistical analyses, confirm that the following items are present in the figure legend, table legend, main text, or Methods section.

n/a	Confirmed	
☐	☒	The exact sample size (*n*) for each experimental group/condition, given as a discrete number and unit of measurement
☐	☒	A statement on whether measurements were taken from distinct samples or whether the same sample was measured repeatedly
☐	☒	The statistical test(s) used AND whether they are one- or two-sided<br>*Only common tests should be described solely by name; describe more complex techniques in the Methods section.*
☐	☒	A description of all covariates tested
☒	☐	A description of any assumptions or corrections, such as tests of normality and adjustment for multiple comparisons
☐	☒	A full description of the statistical parameters including central tendency (e.g. means) or other basic estimates (e.g. regression coefficient) AND variation (e.g. standard deviation) or associated estimates of uncertainty (e.g. confidence intervals)
☐	☒	For null hypothesis testing, the test statistic (e.g. *F*, *t*, *r*) with confidence intervals, effect sizes, degrees of freedom and *P* value noted<br>*Give P values as exact values whenever suitable.*
☒	☐	For Bayesian analysis, information on the choice of priors and Markov chain Monte Carlo settings
☒	☐	For hierarchical and complex designs, identification of the appropriate level for tests and full reporting of outcomes
☒	☐	Estimates of effect sizes (e.g. Cohen's *d*, Pearson's *r*), indicating how they were calculated

Our web collection on statistics for biologists contains articles on many of the points above.

Software and code

Policy information about availability of computer code

Data collection	Immunoblot data were collected using Image Reader LAS-3000 V2.21 software. qPCR data were collected using LC96 Application Software. GVBD timing data of frog oocytes were collected using SPOT Software V5.1. Mouse microscopy data were collected using ZEN 2.1 (black edition).
Data analysis	Band intensities in immunoblots were analysed using Fiji ImageJ and Microsoft Excel 2016. qPCR data were analysed with LC96 Application Software and Microsoft Excel 2016. GVBD timing of frog oocytes was analysed using Fiji ImageJ and Microsoft Excel 2016. Bar graphs were generated using GraphPad Prism 10 software. Statistical analyses were performed using GraphPad Prism 10. Mouse microscopy data were analysed using Fiji ImageJ and ZEN 2 (blue edition) for imaging analysis and data were analyzed using Excel 2016. MS results were analyzed with Spectronaut® v19.6.250122.62635, Perseus v2.1.3.0, Python v3.10.0, R 4.4.2 and R Studio 2024.12.0 including the ggplot2 and ggrepel packages .

For manuscripts utilizing custom algorithms or software that are central to the research but not yet described in published literature, software must be made available to editors and reviewers. We strongly encourage code deposition in a community repository (e.g. GitHub). See the Nature Portfolio guidelines for submitting code & software for further information.

Data

Policy information about availability of data

All manuscripts must include a data availability statement. This statement should provide the following information, where applicable:

- Accession codes, unique identifiers, or web links for publicly available datasets
- A description of any restrictions on data availability
- For clinical datasets or third party data, please ensure that the statement adheres to our policy

The mass spectrometry proteomics data have been deposited to the ProteomeXchange Consortium via the PRIDE72 partner repository with the dataset identifier PXD062261 [https://www.ebi.ac.uk/pride/archive/projects/PXD062261]. The authors declare that all other data generated or analysed during this study are included in this published article (and its supplementary information files). Information and reagents required to repeat the experiments reported in this paper are available from the lead contact upon request. Source data are provided with this paper.

Research involving human participants, their data, or biological material

Policy information about studies with human participants or human data. See also policy information about sex, gender (identity/presentation), and sexual orientation and race, ethnicity and racism.

Reporting on sex and gender	*Use the terms sex (biological attribute) and gender (shaped by social and cultural circumstances) carefully in order to avoid confusing both terms. Indicate if findings apply to only one sex or gender; describe whether sex and gender were considered in study design; whether sex and/or gender was determined based on self-reporting or assigned and methods used.* *Provide in the source data disaggregated sex and gender data, where this information has been collected, and if consent has been obtained for sharing of individual-level data; provide overall numbers in this Reporting Summary. Please state if this information has not been collected.* *Report sex- and gender-based analyses where performed, justify reasons for lack of sex- and gender-based analysis.*
Reporting on race, ethnicity, or other socially relevant groupings	*Please specify the socially constructed or socially relevant categorization variable(s) used in your manuscript and explain why they were used. Please note that such variables should not be used as proxies for other socially constructed/relevant variables (for example, race or ethnicity should not be used as a proxy for socioeconomic status).* *Provide clear definitions of the relevant terms used, how they were provided (by the participants/respondents, the researchers, or third parties), and the method(s) used to classify people into the different categories (e.g. self-report, census or administrative data, social media data, etc.)* *Please provide details about how you controlled for confounding variables in your analyses.*
Population characteristics	*Describe the covariate-relevant population characteristics of the human research participants (e.g. age, genotypic information, past and current diagnosis and treatment categories). If you filled out the behavioural & social sciences study design questions and have nothing to add here, write "See above."*
Recruitment	*Describe how participants were recruited. Outline any potential self-selection bias or other biases that may be present and how these are likely to impact results.*
Ethics oversight	*Identify the organization(s) that approved the study protocol.*

Note that full information on the approval of the study protocol must also be provided in the manuscript.

Field-specific reporting

Please select the one below that is the best fit for your research. If you are not sure, read the appropriate sections before making your selection.

☒ Life sciences ☐ Behavioural & social sciences ☐ Ecological, evolutionary & environmental sciences

For a reference copy of the document with all sections, see nature.com/documents/nr-reporting-summary-flat.pdf

Life sciences study design

All studies must disclose on these points even when the disclosure is negative.

Sample size	No statistical method was used to predetermine sample size. Sample size was determined according to standards in the field.
Data exclusions	No data were excluded from the analysis.
Replication	Experiments were repeated as independent biological replicates. The number of biological replicates for each experiment is indicated in the figure legends.
Randomization	Xenopus laevis oocytes were randomly assigned to experimental groups. Mouse oocytes were randomly assigned to experimental groups.
Blinding	Investigators were not blinded during data collection and analysis.

Reporting for specific materials, systems and methods

We require information from authors about some types of materials, experimental systems and methods used in many studies. Here, indicate whether each material, system or method listed is relevant to your study. If you are not sure if a list item applies to your research, read the appropriate section before selecting a response.

Materials & experimental systems

n/a	Involved in the study	
☐	☒	Antibodies
☒	☐	Eukaryotic cell lines
☒	☐	Palaeontology and archaeology
☐	☒	Animals and other organisms
☒	☐	Clinical data
☒	☐	Dual use research of concern
☒	☐	Plants

Methods

n/a	Involved in the study	
☒	☐	ChIP-seq
☒	☐	Flow cytometry
☒	☐	MRI-based neuroimaging

Antibodies

Antibodies used	4E-TAb1 (c=1µg/ml), 4E-TAb2 (c=1µg/ml), PATL2 (c=1µg/ml), c-Mos (c=0,2µg/ml) and cyclin-B1 (c=0,2µg/ml) antibodies were generated in this study. The following antibodies were purchased from commercial suppliers: Flag-tag antibody (Sigma-Aldrich F1804;1:2000); CPEB1 antibody (Biozol MBS9213514;1:500); ppMAPK antibody (Cell Signaling #9106;1:2000); MAPK antibody (Santa Cruz sc-154;1:2000); Cdk1 antibody (Santa Cruz sc-54; 1:500); p150 antibody (BD Transduction Laboratories 610473;1:2000); DDX6 antibody (for Xenopus samples, Novus Biologicals NB200-191;1:1000); DDX6 antibody (for mouse samples, Abcam ab174277; 1:2000); GFP antibody (Thermo Fisher MA5-15256;1:1000); Puromycin antibody (Sigma-Aldrich MABE343;1:500); eIF4E antibody (Cell Signaling #9742;1:1000); CSDE1 antibody (Bethyl Laboratories A303-158A;1:500); CNOT1 antibody (Cell Signaling #30289;1:1000); 4E-T antibody (for mouse samples, Thermo Fisher Scientific PA5-51680, 1:500); DDB1 antibody (Abcam ab109027; 1:5000); rabbit control antibody for TRIM-Away in Xenopus oocytes (Biozol GSC-A01008). rabbit control antibody for TRIM-Away in mouse oocytes (Merck Millipore 12-370). Myc (9E10) and α-tubulin (DSHB 12G10) antibodies were purified from hybridoma cells. ppCdk (phospho-Thr14 phospho-Tyr15) antibody was a gift from Tim Hunt. ePAB (c=1µg/ml), Zar1l (c=1µg/ml) and Zar2 (c=1µg/ml) antibodies were generated in Heim et al., Development 149, (2022).
Validation	4E-TAb1, 4E-TAb2, PATL2, c-Mos and cyclin-B1 antibodies were validated in this study. ePAB, Zar1l and Zar2 antibodies were validated in Heim et al., Development 149, (2022). Flag-tag (Sigma-Aldrich F1804), GFP (Thermo Fisher MA5-15256) and Puromycin (Sigma-Aldrich MABE343) antibody validation can be found on the company website. DDX6 (Abcam ab174277), DDB1 (Abcam ab109027) and 4E-T (Thermo Fisher Scientific PA5-51680) antibody validation can be found on the company website and they were previously used in Cheng et al., Science 378, (2022). CPEB1 (Biozol MBS9213514), ppMAPK (Cell Signaling #9106), MAPK (Santa Cruz sc-154), Cdk1 (Santa Cruz sc-54), p150 (BD Transduction Laboratories 610473), DDX6 (Novus Biologicals NB200-191) antibody validation can be found on the company website and they were previously used in Heim et al., Development 149, (2022). ppCdk antibody was previously used e.g. in Bouftas et al., Dev Cell 57, (2022) and Heim et al., Development 149, (2022). eIF4E (Cell Signaling #9742); CSDE1 (Bethyl Laboratories A303-158A) and CNOT1 (Cell Signaling #30289) antibodies target highly conserved regions of the respective proteins and validation can be found on the company website. Myc and Tubulin antibodies were generated from established hybridoma cell lines and previously used in A. Heim et al., EMBO Rep 19, (2018) and Heim et al., Development 149, (2022).

Animals and other research organisms

Policy information about studies involving animals; ARRIVE guidelines recommended for reporting animal research, and Sex and Gender in Research

Laboratory animals	Female Xenopus laevis frogs and 7- to 10-week-old C57BL/6N mice. Mice were housed at 21°C ambient temperature, 52–55% humidity and a 14-h light/10-h dark cycle.
Wild animals	No wild animals were used in this study.
Reporting on sex	Oocytes were collected from female Xenopus laevis frogs and female mice.
Field-collected samples	No field-collected samples were used in this study.
Ethics oversight	All procedures performed with Xenopus laevis frogs were approved by the Regional Commission, Freiburg, Germany (Az. 35-9185.81/G-17/121 and 35-9185.81/G-22/080). Maintenance and handling of all mice were carried out in the animal facility of Max Planck Institute for Multidisciplinary Sciences according to international animal welfare rules (Federation for Laboratory Animal Science Associations guidelines and recommendations). Requirements of formal control of the German national authorities and funding organizations were satisfied, and the study received approval by the Niedersächsisches Landesamt für Verbraucherschutz und Lebensmittelsicherheit (LAVES).

Note that full information on the approval of the study protocol must also be provided in the manuscript.

Plants

Seed stocks

Report on the source of all seed stocks or other plant material used. If applicable, state the seed stock centre and catalogue number. If plant specimens were collected from the field, describe the collection location, date and sampling procedures.

Novel plant genotypes

Describe the methods by which all novel plant genotypes were produced. This includes those generated by transgenic approaches, gene editing, chemical/radiation-based mutagenesis and hybridization. For transgenic lines, describe the transformation method, the number of independent lines analyzed and the generation upon which experiments were performed. For gene-edited lines, describe the editor used, the endogenous sequence targeted for editing, the targeting guide RNA sequence (if applicable) and how the editor was applied.

Authentication

Describe any authentication procedures for each seed stock used or novel genotype generated. Describe any experiments used to assess the effect of a mutation and, where applicable, how potential secondary effects (e.g. second site T-DNA insertions, mosiacism, off-target gene editing) were examined.
